# Effect of Fuzheng Huayu capsule combined with Pegasys on genotype 1 hepatitis C fibrosis and cell apoptosis

**DOI:** 10.3892/etm.2014.1891

**Published:** 2014-08-08

**Authors:** BO ZHANG, MINTAO HU, LIHUA HUANG, YUNCHUAN PU, HAO PEI, ZHONG HUA, SHANGZHI YAO

**Affiliations:** Department of Infectious Diseases, The Fifth People’s Hospital of Wuxi, Wuxi, Jiangsu 214005, P.R. China

**Keywords:** Fuzheng Huayu capsule, Pegasys, rat model, liver fibrosis

## Abstract

The aim of the present study was to observe the effects of Fuzheng Huayu capsule combined with Pegasys (peginterferon α-2a) on hepatic fibrosis in rats and in the treatment of patients with genotype 1 hepatitis C and hepatic cirrhosis. A dimethylnitrosamine (DMN)-induced rat model of liver injury was established. Fuzheng Huayu capsule combined with Pegasys was administered to the rats and the DMN-induced hepatocyte apoptosis was observed. In addition, a total of 100 patients with genotype 1 hepatitis C and hepatic cirrhosis were treated by oral administration of Fuzheng Huayu capsule combined with Pegasys or with Pegasys alone. The therapeutic effect of Fuzheng Huayu capsule combined with Pegasys was analyzed. Following the oral administration of Fuzheng Huayu capsule combined with Pegasys to the DMN model rats, the expression of α-smooth muscle actin was found to be significantly reduced, hemopoietic stem cell apoptosis was increased and liver cell apoptosis was reduced. These indices were significantly different compared with those in the model group (P<0.05). Liver function and liver fibrosis were markedly recovered in hepatitis C patients with hepatic cirrhosis following treatment with the combination treatment compared with those in the patients treated with Pegasys alone (P<0.05). In conclusion, the combination of Fuzheng Huayu capsule with Pegasys inhibited liver fibrosis and cell apoptosis, and may be a novel therapeutic strategy for the treatment of patients with compensated cirrhosis due to hepatitis C. This study provides a method for the optimization of existing treatment strategies and for the establishment of potentially effective combination therapies.

## Introduction

Hepatitis C is an infectious disease that is prevalent around the world. Hepatitis C virus (HCV) induces chronic infection in ~80% of infected individuals. Approximately 10–20% of patients develop cirrhosis and nearly 5% will eventually suffer from liver cancer (1). The aim of HCV treatment is to clear the virus and the symptoms associated with hepatitis C infection (2,3). At present, combined treatment with pegylated interferon α (PEG-IFN) and the antiviral drug ribavirin is the standard approach for HCV treatment (4–6). In a previous study, the sustained virological response (SVR) was found to be 8, 15 and 30% in patients with chronic hepatitis C and cirrhosis following treatment with IFN, 90 μg PEG-IFN and 180 μg PEG-IFN, respectively (7). In the present study, the effect of Fuzheng Huayu capsule combined with Pegasys (peginterferon α-2a) was investigated on cell apoptosis in rats with dimethylnitrosamine (DMN)-induced liver injury and on liver function and fibrosis in patients with hepatitis C and hepatic cirrhosis. These results may provide a novel therapeutic strategy for the treatment of patients with hepatic cirrhosis.

## Materials and methods

### Animals

A total of 60 Wistar male rats (200±10 g) were provided by Jiangsu Laboratory Animal Center (Suzhou, China). This study was performed in accordance with the recommendations in the Guide for the Care and Use of Laboratory Animals of the National Institutes of Health (8th edition, Bethesda, MD, USA, 2010). The animal use protocol was reviewed and approved by the Institutional Animal Care and Use Committee of the Fifth People’s Hospital of Wuxi (Wuxi, China). The rats were randomly divided into two groups, a normal control group (n=20) and a model group (n=40). After four weeks, the model group was subdivided into a model control group and a treatment group (Fuzheng Huayu capsule combined with Pegasys). The rats were administered a gavage of distilled water or a solution of the combined drugs [4.5 g Fuzheng Huayu (6 capsules) and 180 μg Pegasys in 10 ml distilled water] respectively, at a dosage of 10 ml/kg, six times a week for four weeks. Blood was then taken from the inferior vena cava and the serum was obtained through centrifugation. In addition, liver tissues were prepared for further experiments.

The model group was intraperitoneally injected with 0.5% DMN (2 ml/kg). The injections were administered on three successive days with two-thirds of a full dose and then no injections were administered for 4 days. This was continued for four weeks and the last two injections were performed with one shot of two-thirds of a full dose and one shot of half of a full dose, respectively. An equal volume of saline was injected in the normal group. Once the DMN model was established, two rats were randomly sacrificed and the pathological condition of the liver was analyzed using an α-SMA monoclonal antibody (Dako, Agilent Technologies, Santa Clara, CA, USA). The model was successfully established since liver fibrosis was observed. Following fixation of the liver tissues with 10% formalin, dehydration, transparency and wax immersion, cell apoptosis indices [apoptotic hemopoietic stem cell (HSC), apoptotic hepatocyte and α-smooth muscle actin (SMA) levels] were analyzed using terminal deoxynucleotidyl transferase dUTP nick end labeling (TUNEL; Roche, Basel, Switzerland), α-SMA and Masson triple staining (both from Shanghai Bogoo Biotechnology Co., Ltd., Shanghai, China). These experimental procedures were performed in accordance with the manufacturers’ instructions.

### Patients

A total of 100 patients with genotype 1 hepatitis C and hepatic cirrhosis from the Fifth People’s Hospital of Wuxi (Wuxi, China) were analyzed. The patients were randomly divided into a treatment group (group A) and a control group (group B) with 50 patients in each group. In the treatment group, there were 28 males and 22 females, aged 42.3±10.2 years old and with a disease course of 5.7±1.1 years. In the control group, there were 26 males and 24 females aged 40.3±3.4 years old and with a disease course of 7.2±2.2 years. All patients were HCV antibody positive, had an HCV RNA level of ≥500 cps/ml and an Ishak fibrosis score of 2, 3 or 4 (8). This study was performed in accordance with the Declaration of Helsinki and with approval from the Ethics Committee of the Fifth People’s Hospital of Wuxi. Written informed consent was obtained from all participants.

### Clinical trials

The two groups of patients were treated with 180 μg Pegasys (Shanghai Roche Pharmaceuticals Co., Ltd, Shanghai, China) combined with 1,000–1,200 μg ribavirin for 48 weeks. The patients in group A were also treated with a 4.5 g Fuzheng Huayu capsule (Shanghai Yellow Sea Pharmaceutical Co., Ltd., Shanghai, China) each day. The patients in group B were given a placebo. The levels of aminotransferase (ALT), total bilirubin (TBiL), aspartate aminotransferase (AST), albumin, α-fetoprotein (AFP) and total bile acids (TBA) were determined using a Beckman-Coulter 3XL flow cytometry instrument (Beckman-Coulter, Miami, FL, USA), a CEQ-8000 sequencer instrument (Beckman-Coulter, Miami, FL, USA), a PE-9600 PCR instrument (Perkin-Elmer, Fremont, CA, USA) and an 7600 automatic biochemistry analyzer (Hitachi Ltd., Tokyo, Japan). Liver fibrosis indicators, including serum hyaluronic acid (HA), laminin (LN), type IV collagen (IVC) and type III procollagen (PCIII) were detected using an ELISA kit (Euroimmun, Lübeck, Germany) and a TUNEL apoptosis kit (Roche, Basel, Swiss). Fas and FasL levels of PBMCs were detected with a Fas and FasL testing kit (Shenzhen Jingmei Biotech Co., Ltd., Shenzhen, China) according to the manufacturer’s instructions.

### Statistical analysis

The quantitative data was presented as the mean ± standard deviation and analyzed using SPSS software, version 16.0 (SPSS Inc., Chicago, IL, USA). Statistical analysis was performed using a Student’s t-test for comparison and a Spearman’s rank correlation coefficient. P<0.05 was considered to indicate a statistically significant difference.

## Results

### Animal results

The TUNEL, α-SMA and Masson staining results revealed that there were no apoptotic HSCs in the normal group. However, in DMN rats, a number of apoptotic HSCs were observed around the hepatic sinusoid. The number of apoptotic HSCs was greater in the DMN model rats than in the DMN group treated with Fuzheng Huayu capsule combined with Pegasys. A small number of hepatocytes became apoptotic in the normal group, while there was a significant increase in the number of apoptotic hepatocytes in the DMN group. The number of apoptotic cells was markedly reduced in the rats treated with Fuzheng Huayu capsule combined with that in the model rats (Table I).

### Clinical observations

**Figure 1:**
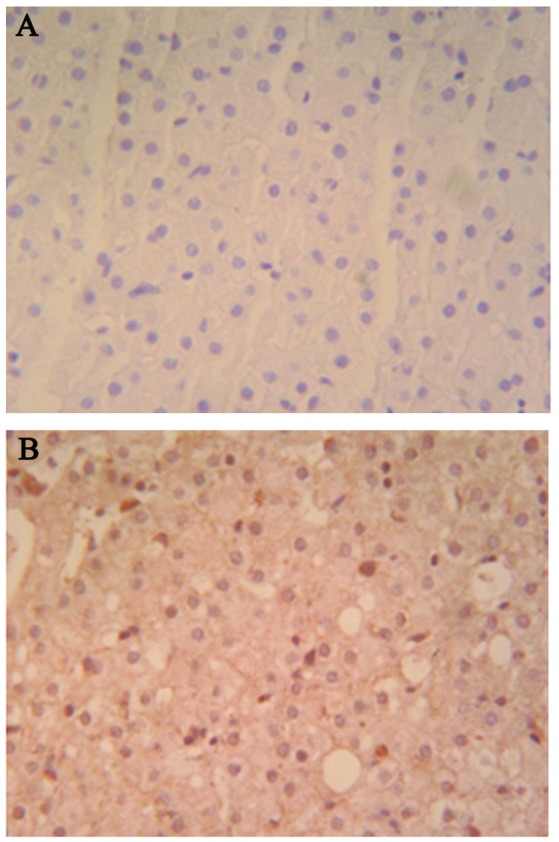
Liver tissue pathology following treatment with (A) Pegasys alone and (B) Fuzheng Huayu and Pegasys (magnification, ×400). Hematoxylin-eosin staining was used.

Significant differences in ALT, TBiL, AST, ALB, AFP and TBA were observed between the patients treated with Fuzheng Huayu capsule combined with Pegasys and those treated with Pegasys alone (P<0.01; Table II). In addition, the levels of markers for hepatic fibrosis, including HA, LN, IVC and PCIII were significantly reduced in the combined treatment group (P<0.01; Table III). These results indicate that Fuzheng Huayu capsule combined with Pegasys improves the efficacy of Pegasys in protecting the liver from fibrosis. The Fas and FasL levels in PBMCs were detected in the blood. The expression of Fas and FasL was attenuated following treatment with Fuzheng Huayu capsules (Table IV). The results demonstrate that the function of the immune system was influenced by liver fibrosis following treatment. This suggests that liver injury and hepatocyte apoptosis were significantly improved following treatment with Fuzheng Huayu capsule combined with Pegasys, as indicated by liver tissue pathology (Fig. 1).

## Discussion

Chronic liver fibrosis is a common pathological process in various chronic liver diseases. If chronic hepatitis C is not treated effectively, it increases the risk of liver cirrhosis and cancer (9). The treatment and prevention of liver fibrosis has been investigated clinically. Fuzheng Huayu capsule has been shown to alleviate blood stasis and have a beneficial effect on the liver (10). In the present study, the effect of Fuzheng Huayu capsule combined with Pegasys was investigated by analyzing liver indicators following treatment.

Hepatocyte apoptosis was found to be significantly reduced in rats with DMN-induced liver injury following treatment with Fuzheng Huayu capsule combined with Pegasys. The decreased activation of HSCs, alleviation of endothelial cell damage and reduction of hepatocytes may be involved in this process. Reduced secretion of TGF-β and TNF-α, which promote the hepatocyte apoptosis, may be important for the function of these combined drugs (11,12). These results demonstrated that Fuzheng Huayu capsule combined with Pegasys improved liver fibrosis induced by hepatitis C, which are consistent with the previously reported studies (13–15). Fuzheng Huayu capsule includes: *Salviae miltiorrhizae,* which promotes blood circulation and alleviates blood stasis, as well as cooling and nourishing the blood; peach seed, which improves ‘circulation of qi’, alleviates blood stasis and activates enzymes required in bilirubin metabolism; *Gynostemma pentaphyllum*, which ‘replenishes qi’ to invigorate the spleen and regulates the immunity function; *Cordyceps sinensis*, which ‘tonifies the spleen’ and strengthens humoral immunity; and *Schisandra*, which ‘supplements qi’ and promotes the production of body fluid to increase the function of cell immunity (16,17,18). The metabolism of CCl_4_ is also inhibited by *Schisandra* to protect hepatocytes (19). Based on this, Fuzheng Huayu capsule combined with Pegasys was used in the present study for the treatment of liver fibrosis, and it was hypothesized that the drug would improve the blood flow to the liver, strengthen liver detoxification and enhance hepatocyte regeneration.

In the present study, hepatocyte apoptosis was investigated in a DMN-induced rat model of liver fibrosis and clinical patients following treatment with Fuzheng Huayu capsule combined with Pegasys. The results demonstrate that there was a significant improvement in cell apoptosis following the treatment. The observed effects indicate that Fuzheng Huayu capsule and Pegasys efficiently enhance the body’s immune function and promote liver regeneration (20–22). There are two aspects of liver fibrosis treatment: etiological treatment of the primary disease and treatment of liver fibrosis itself. The Fuzheng Huayu capsule combined with Pegasys treated the two aspects of the disease and decreased serum fibrosis indexes to inhibit or delay the development of liver fibrosis. This is the first study, to the best of our knowledge, to use the Fuzheng Huayu capsule combined with Pegasys for the treatment of patients with hepatitis C and hepatic cirrhosis. The clinical results demonstrate that the combined drugs exert certain curative effects in patients with hepatitis C and increase their quality of life. In conclusion, the present study provides a method for the optimization of existing treatment strategies and for the establishment of potentially effective combination therapies.

## Figures and Tables

**Table I:** Number of apoptotic HSCs and hepatocytes and the expression of α-SMA in the three groups of rats.

Group	Apoptotic HSCs (per high power field, magnification, ×40)	Apoptotic hepatocytes (per high power field, (magnification, ×40)	α-SMA (percentage of positive area)
Normal	0	0.95±0.27	1.46±0.13
Model	0.15±0.05	3.22±0.25	8.42±0.53
Treatment	0.27±0.08	2.49±0.17	4.11±0.45
F-value	627.5	2.51	0.12
P-value	<0.05	<0.05	<0.05

Values are presented as the mean ± standard deviation. HSCs, hemopoietic stem cells; SMA, smooth muscle actin.

**Table II:** Liver function following treatment with Fuzheng Huayu and Pegasys (treatment) or Pegasys alone (control).

Group	ALT (U/l)	TBiL (μmol/l)	AST (U/l)	ALB (G/l)	AFP (ng/ml)	TBA (μmol/l)
Control	88.65±20.75	37.85±9.65	213.48±44.61	25.29±1.65	56.17±12.91	52.89±21.61
Treatment	33.21±14.65	12.38±6.45	65.26±21.47	39.46±2.47	19.23±5.63	89.34±11.78
t-value	4.30	2.78	4.51	5.12	8.56	4.24
P-value	<0.01	<0.01	<0.01	<0.01	<0.01	<0.01

Values are presented as the mean ± standard deviation. ALT, aminotransferase; TBiL, total bilirubin; AST, aspartate aminotransferase; ALB, albumin; AFP, α-fetoprotein; TBA, total bile acids.

**Table III:** Serum fibrosis indices following treatment with Fuzheng Huayu and Pegasys (treatment) or Pegasys alone (control).

Group	HA (μg/l)	LN (μg/l)	IVC (μg/l)	PCIII (μg/l)
Control	287.04±67.27	145.38±67.35	135.19±68.79	202.52±49.87
Treatment	245.38±52.19	122.73±71.19	110.32±42.16	177.27±72.26
t-value	4.77	3.87	4.65	7.67
P-value	<0.05	<0.05	<0.05	<0.05

Values are presented as the mean ± standard deviation. HA, hyaluronic acid; LN, laminin; IVC, type IV collagen; PCIII, type III procollagen.

**Table IV:** Expression of Fas and FasL in the peripheral blood mononucleated cells of patients following treatment with Fuzheng Huayu and Pegasys (treatment) or with Pegasys alone (control).

Group	Fas	FasL
Control	0.69±0.25	0.82±0.17
Treatment	0.42±0.15	0.57±0.19
t-value	6.43	4.78
P-value	<0.05	<0.05

Values are presented as mean ± standard deviation. FasL, Fas ligand.
